# Positive Youth Development Programs Targeting Students with Greater Psychosocial Needs: Subjective Outcome Evaluation

**DOI:** 10.1100/tsw.2008.3

**Published:** 2008-01-14

**Authors:** Daniel T. L. Shek, Tak Yan Lee, Rachel C.F. Sun, Daniel W.M. Lung

**Affiliations:** ^1^ Centre for Quality of Life, Hong Kong Institute of Asia-Pacific Studies, The Chinese University of Hong Kong, Hong Kong; ^2^ Kiang Wu Nursing College of Macau, Macau; ^3^ Social Welfare Practice and Research Centre, The Chinese University of Hong Kong, Hong Kong; ^4^ Department of Applied Social Studies, City University of Hong Kong, Hong Kong

**Keywords:** subjective outcome evaluation, positive youth development, adventure-based counseling approach, volunteer training and services

## Abstract

The Tier 2 Program of the Project P.A.T.H.S. (Positive Adolescent Training through Holistic Social Programmes) targets adolescents with greater psychosocial needs, and the related programs were designed and implemented by school social workers. After completion of the Tier 2 Program, 2,173 students in 52 schools responded to the Subjective Outcome Evaluation Form (Form C), assessing their views of the program, instructors, and perceived effectiveness of the program. Based on the consolidated reports submitted by the agencies to the funding body, the research team aggregated the consolidated data to form a “reconstructed” overall profile of the perceptions of the program participants. Four major types of program were identified, including programs based on the adventure-based counseling approach (N = 8), programs concentrated on volunteer training and services (N = 7), programs incorporating both adventure-based counseling and volunteer training elements (N = 30), and other programs with different foci (N = 7). Results showed that high proportions of the respondents had positive perceptions of the programs and the instructors, and roughly four-fifths of the respondents regarded the program as helpful to them. The present study provides support for the effectiveness of the Tier 2 Program of P.A.T.H.S. in Hong Kong for the experimental implementation phase.

### INTRODUCTION

The Project "P.A.T.H.S. to Adulthood: A Jockey Club Youth Enhancement Scheme" is a large-scale, positive youth development program designed for junior secondary school students (Secondary 1 to 3, i.e., Grade 7 to 9) in Hong Kong[1]. “P.A.T.H.S.” denotes Positive Adolescent Training through Holistic Social Programmes. It consists of two tiers of programs. The Tier 1 Program targets all students joining the program in a particular form (i.e., universal prevention initiative). Through the use of structured curriculum, students learn competencies with reference to the 15 positive youth development constructs[2]. The Tier 2 Program is specially designed for students with greater psychosocial needs in different psychosocial domains (i.e., selective prevention initiative). It is noteworthy that the researchers deliberately avoided using the term “at risk” because the term is very stigmatizing in the Chinese culture, and it deters parents and students from joining the related programs. Based on the developmental needs of the students in a particular school, the school social work service providers design and implement the Tier 2 Program.

There are several characteristics of the Tier 2 Program in the project. First, the Tier 2 Program (Selective Program) targets adolescents with greater psychosocial needs who are identified in the Tier 1 Program and/or via other sources. It is recommended that information based on multiple sources, including objective assessment tools (e.g., Family Assessment Instrument, Life Satisfaction Scale, Hong Kong Student Information Form), teachers’ ratings, student records, and other relevant quantitative and qualitative information based on systematic assessment, will be used to identify students for the Tier 2 Program. Second, it is suggested that particular attention should be paid to students with special needs in the academic, personal (e.g., adjustment, mental health, and value concerns), interpersonal, and family domains. As a general guideline, it is expected that at least one-fifth of the adolescents and/or their parents of the Tier 1 participants will participate in the Tier 2 Program. However, under some special circumstances (e.g., many students have value confusions over material possession and difficulty in financial management), Tier 2 Program targeting more than one-fifth of the students at each form could be designed within the available financial resources. Based on the above general guidelines, for those schools providing the full Tier 1 Program (i.e., 40 curriculum units in a 20-h program), they were expected to serve at least one-fifth of the students at each form. For those schools providing the core Tier 1 Program (i.e., 20 curriculum units in a 10-h program), they would have to serve at least two-fifths of the students at each form.

For students who are identified as having special needs, they and/or their families will receive services based on the Tier 2 Program. To create more flexibility for the workers and in view of the diverse needs of the students, the NGO applicants have the choice of designing appropriate programs that target the needs of the students with reference to the positive youth development constructs, goals, and objectives covered in this project. Several nonmutually exclusive examples for the Tier 2 Program include (a) mentorship programs involving the alumni of the schools, (b) mental health promotion programs, (c) adventure-based counseling, (d) parenting programs, (e) service learning programs, and (f) resilience enhancement programs.

This paper presents and discusses the findings of an evaluation of the Tier 2 Program implemented in the experimental implementation phase of the project in 2005/2006 based on the perspective of the participants. Although it is very common for social work agencies to design programs for adolescents with greater psychosocial needs (e.g., adventure-based counseling, voluntary training program), systematic evaluation and documentation of program evaluation have been rarely found in the local social work literature[3]. This paper presents a systematic study on social work intervention with adolescents. Besides giving some thoughts on the quality of the program delivered, it was also hoped that this paper would contribute to the development of evidence-based social work practice in Hong Kong. Actually, in the literature on positive youth development programs, many programs have been designed to help adolescents with greater psychosocial needs and the related evaluation findings have been documented[4,5,6].

## METHODS

### Participants and Procedures

A total of 52 schools joined the experimental implementation phase of the project in 2005/2006. In these schools, there were 3,072 participants involved in the Tier 2 Program, of which 2,718 students were identified by teachers, parents, and/or self-administered questionnaires as having greater psychosocial needs and were invited to join the Tier 2 Program. The remaining 354 participants were the parents and teachers of those identified students and they were also invited to participate in the Tier 2 Program. The mean number of participants joining the Tier 2 Program per school was 59.08 (range: 21–274). The average number of sessions provided per school (normally 1.5–3 h per session) was 19.53 (range: 1–63).

The participants were invited to respond to a subjective outcome evaluation questionnaire usually immediately after completion of the Tier 2 Program. A total of 2,173 participants (mean = 41.79 participants per school; range: 20–151) responded to the Subjective Outcome Evaluation Form (Form C) developed by the research team[7]. The overall response rate was 70.74%. There are three possible reasons for this response rate: (1) some participants withdrew from the Tier 2 Program before its termination, (2) some participants were absent in the last session and did not complete the evaluation form, and (3) some schools did not invite the adult participants to complete the evaluation questionnaire because the adults had joined a very small proportion (<10%) of the program.

Before the evaluation data were collected, the purpose of the evaluation was explained and the principle of confidentiality was emphasized repeatedly. The participants were asked to indicate their wish if they did not want to respond to the evaluation questionnaire (i.e., "passive" informed consent from the students were obtained). All participants responded to all scales in the evaluation form in a self-administration format. Adequate time was provided for the participants to complete the questionnaire. To facilitate the program evaluation, the research team developed an evaluation manual with standardized instructions for collecting the subjective outcome evaluation data[7]. In addition, adequate training was provided to the social workers during the 20-h training workshops on how to collect and analyze the data using Form C.

### Instruments

The Subjective Outcome Evaluation Form (Form C) was designed by Daniel Shek and Andrew Siu[7], with the aim to measure the perceptions of the Tier 2 Program. There are seven parts in this evaluation form:

- Participants' perceptions of the program, such as program design, quality of service, appropriateness of the program, and interaction among the participants (8 items).
- Participants' perceptions of the workers, such as the preparation of the workers, professional attitude and knowledge, and interaction with the participants (8 items).
- Participants' perception of the effectiveness of the program, such as promotion of different psychosocial competencies, resilience, and overall personal development (8 items).
- Things that the participants appreciated most (open-ended question).
- Opinion about the workers (open-ended question).
- Things that the participants learned from the program (open-ended question).
- Areas that require improvement (open-ended question).

After collecting the data, the social work service providers were requested to input the data in an EXCEL file developed by the research team that would automatically compute the frequencies and percentages associated with the different ratings for an item. When the providers submitted the reports, they were also requested to submit the soft copy of the consolidated data sheets. The data from all service providers were then aggregated to “reconstruct” the overall profile. Since some amendments were made during the program implementation (e.g., program cancellation due to insufficient participants), the delivered program content was reported again in the program report.

Since the program contents of the Tier 2 Program were designed according to the various needs and the contextual situations of different schools, it is hard to compare their results directly. Therefore, content analysis on the program contents was conducted by identifying the major program elements of the Tier 2 Program for all participating schools. Two trained research assistants (one with a Bachelor degree in Social Work and one with a Bachelor degree in Psychology) categorized the program contents delivered by the social workers as indicated in the program reports submitted to the research team. The criteria were strictly set to categorize a program according to the program proposal or revised proposal together with the actual activities.

### RESULTS

Results of the content analyses showed that three major types of program elements were identified, namely, the adventure-based counseling approach, volunteer training and services, and others. A program will be classified as containing the adventure-based activities and counseling (ABC) elements if and only if the espoused theory was matched with the theory-in-action[8]. The most frequently found activities related to volunteer training and services were program planning skills and organizing volunteer services. The most popular volunteer services were visiting the elderly and services for mentally challenged people. The programs without elements of ABC or volunteer training and services were classified as “others”. Examples of this category included groups promoting self-understanding, social skills training, and community-based oral history projects. An analysis of the programs delivered by the social workers showed that two types of program approaches were frequently adopted in the Tier 2 Program. They were volunteer training and services, and the use of the ABC approach. Over 85% of the Tier 2 Program delivered by 52 secondary schools contained at least one of these two elements.

The characteristics of the Tier 2 Program are presented in Table 1. The Tier 2 Program can be categorized into four types by the main types of program elements used or program approaches adopted. They are (1) the ABC approach together with volunteer training and services (Type A programs: 57.7%), (2) ABC only (Type B program: 15.4%), (3) volunteer training and services only (Type C program: 13.5%), and (4) other approaches (Type D program: 13.5%). For the Type D programs, programs included promoting discipline through military march training, social skills training, promoting learning skills and motivation, promoting self-understanding, and community-based oral history projects.

The ABC approach and volunteer training and services were the two major program elements adopted for the Tier 2 program. The mean overall effectiveness of all programs ranged from 4.46 to 4.86 on a 6-point scale towards the positive side. P.A.T.H.S. made use of 15 constructs identified by Catalano and colleagues[9]. The average number of constructs indicated in the reports for each type of programs ranged from 3.33 to 10 and the mode was 4 (Table 1).

Based on the participants’ answers to the closed-ended questions (quantitative data), there are several observations. First, over 80% of the respondents perceived the Tier 2 Program in which they participated in a positive manner (Table 2). For example, 86.82% of the participants indicated that they were satisfied with the service and 85.28% felt that the service delivered could achieve the planned objectives. Second, a very high proportion of the participants had a positive evaluation of the social workers (Table 3). For example, 87.29% of the respondents indicated that the worker(s) had professional knowledge, 88.81% of the respondents indicated that worker(s) were well prepared for the program, and nearly 90% of participants revealed that they were satisfied with the worker(s). Third, as shown in Table 4, roughly four-fifths of the respondents perceived that the program had induced positive changes in them. For example, the participants learned how to help themselves through participating in the program (86.11%), perceived the services enhanced their growth (86%), and they learned how to solve their problems through participating in the program (85.67%). Fourth, it was interesting to find that 82.01% of the participants would recommend the program to their friends with similar needs (Table 2) and a similar percentage of them (82.68%) indicated that they would join similar programs in future (Table 4). Finally, as indicated in Table 4, roughly four-fifths of the respondents indicated that their behavior became better (82.21%) and they had positive changes(s) after joining the program (84.94%).

**TABLE 1**
**Summary Program Characteristics and Effectiveness**

| Main Program Approach | Clientele | Average No. of Participants | Average Program Attendance (%) | Average No. of Program Aims Indicated in the Reports | Average No. of Constructs Indicated in the Reports | Mean of Overall Effectiveness |
|---|---|---|---|---|---|---|
| ABC approach and volunteer training and services (Type A) (N = 30) | a (N = 25) | 65.12 | 83.34 | 1.96 | 4.81 | 4.46 |
| | b (N = 3) | 107 | 86.63 | 1.33 | 10 | 4.86 |
| | c (N = 2) | 137 | 84.65 | 2 | 3.50 | 4.63 |
| ABC approach only (Type B) (N = 8) | a (N = 6) | 53 | 78.86 | 2.33 | 3.33 | 4.52 |
| | b (N = 1) | 77 | 53 | 2 | 4 | 4.59 |
| | c (N = 1) | 31 | 100 | 1 | 4 | 4.50 |
| Volunteer training and services only (Type C) (N = 7) | a (N = 7) | 76.71 | 71.29 | 4.86 | 5.67 | 4.57 |
| Other approaches (Type D) (N = 7) | a (N = 7) | 37 | 83.80 | 1.86 | 4.14 | 4.76 |

a, Students involved; b, students and parents involved; c, students, parents, and teachers involved.

In comparing the four different types of programs, it was found that Type D programs received the largest number of positive ratings from the participants: seven out of eight items on the program in Table 2 (binomial test, $p < 0.05$), five out of eight items on the worker in Table 3 (binomial test, $p < 0.05$), and all items on program effectiveness in Table 4 (binomial test, $p < 0.01$). On the other hand, all the ratings of the Type A program were the lowest among the four types of the Tier 2 Program (binomial test, $p < 0.01$ in all cases), although all the ratings were positive and ranged from 77.99 to 86.44% (Tables 2 to 4).

### DISCUSSION

Several points can be highlighted in the study. First, Types B (ABC approach only) and C (volunteer training and services only) programs each took up about 14–15% of all programs, but the Type A program (a mixture of Types B and C) took up 57.7%. These two program approaches covered 86.5% of all delivered programs and were the dominant approaches adopted. They were both popular program approaches used by social workers working with adolescents and young people in Hong Kong. Both of them ground their theoretical base in the experiential learning theory[6,10,11]. The Type C program has a much longer history that can date back to the late 1960s and has since been a popular program, while Type B was formally adopted as the major program theory for a huge social intervention program entitled

"The Understanding the Adolescent Project" (UAP) to combat problems among students identified as adolescent-at-risk from 2001 to 2004 in Hong Kong[12,13].

It is noteworthy that although the ABC approach and volunteer training and services are popular programs offered by social workers in Hong Kong, the effectiveness of these programs has not been properly documented. Although the ABC approach can help to instill novel experiences in young people who are usually not good at verbal expression (i.e., walk therapy rather than talk therapy) and its usefulness has been endorsed by counselors[14,15,16], its long-term effect is questionable[6]. Therefore, the present findings constitute interesting additions to the literature.

**TABLE 2**
**Comparison of the Positive Views Towards Tier 2 Program Among Different Program Approaches**

| | Participants with Positive Responses in Different Program Approach | | | | | | | | | |
|---|---|---|---|---|---|---|---|---|---|---|
| | ABC Approach and Volunteer Training and Services[1] | | ABC Approach[2] | | Volunteer Training and Services[3] | | Others[4] | | Overall | |
| | N (Total Response) | % | N (Total Response) | % | N (Total Response) | % | N (Total Response) | % | N (Total Response) | % |
| The activities were carefully planned. | 1039 (1298) | 80.05 | 299 (331) | 90.33 | 269 (307) | 87.62 | 214 (233) | 91.85 | 1821 (2169) | 83.96 |
| The quality of the service was high. | 1030 (1297) | 79.41 | 296 (329) | 89.97 | 268 (307) | 87.30 | 208 (233) | 89.27 | 1802 (2166) | 83.19 |
| The service provided could meet the participants' needs. | 1021 (1295) | 78.84 | 283 (330) | 85.76 | 273 (307) | 88.93 | 208 (230) | 90.43 | 1785 (2162) | 82.56 |
| The service delivered could achieve the planned objectives. | 1063 (1297) | 81.96 | 297 (331) | 89.73 | 275 (307) | 89.58 | 213 (232) | 91.81 | 1848 (2167) | 85.28 |
| Participants could get the service they wanted. | 1010 (1295) | 77.99 | 287 (331) | 86.71 | 259 (306) | 84.64 | 208 (229) | 90.83 | 1764 (2161) | 81.63 |
| Participants had much interaction with other participants. | 1065 (1293) | 82.37 | 289 (328) | 88.11 | 259 (307) | 84.36 | 206 (232) | 88.79 | 1819 (2160) | 84.21 |
| Participants would recommend others who have similar needs to participate in the program. | 1021 (1291) | 79.09 | 279 (327) | 85.32 | 266 (307) | 86.64 | 203 (232) | 87.50 | 1769 (2157) | 82.01 |
| On the whole, participants were satisfied with the service. | 1090 (1292) | 84.37 | 294 (323) | 91.02 | 273 (307) | 88.93 | 213 (232) | 91.81 | 1870 (2154) | 86.82 |

[1] The program contents related to both the ABC approach and volunteer training and services were indicated in the Tier 2 Program reports.

[2] The program contents related to the ABC approach were indicated in the Tier 2 Program reports.

[3] The program contents related to volunteer training and services were indicated in the Tier 2 Program reports.

[4] Except the ABC approach and volunteer training and services, other program contents were indicated in the Tier 2 Program reports.

The positive evaluation findings based on the service learning programs are basically consistent with the literature that services that engagement in voluntary services is conducive to the positive development of adolescents[17,18]. However, because students in Hong Kong may treat voluntary work as a vehicle for enriching their resumes, there is a need to understand their motives for joining voluntary services and the long-term effects of engaging in voluntary work in Hong Kong. In short, in view of the paucity of the research findings for these two categories of services in Hong Kong, the present findings contribute to the database on program evaluation in the Chinese context.

**TABLE 3**
**Comparison of the Positive Views Towards Tier 2 Program Workers Among Different Program Approaches**

| | Participants with Positive Responses in Different Program Approach | | | | | | | | | |
|---|---|---|---|---|---|---|---|---|---|---|
| | ABC Approach and Volunteer Training and Services | | ABC Approach | | Volunteer Training and Services | | Others | | Overall | |
| | N (Total Response) | % | N (Total Response) | % | N (Total Response) | % | N (Total Response) | % | N (Total Response) | % |
| The worker(s) had professional knowledge. | 1094 (1296) | 84.41 | 303 (329) | 92.10 | 275 (307) | 89.58 | 216 (231) | 93.51 | 1888 (2163) | 87.29 |
| The worker(s) demonstrated good working skills. | 1076 (1297) | 82.96 | 298 (329) | 90.58 | 275 (308) | 89.29 | 215 (232) | 92.67 | 1864 (2166) | 86.06 |
| The worker(s) were well prepared for the program. | 1114 (1294) | 86.09 | 309 (328) | 94.21 | 280 (308) | 90.91 | 218 (233) | 93.56 | 1921 (2163) | 88.81 |
| The worker(s) understood the needs of the participants. | 1065 (1295) | 82.24 | 297 (327) | 90.83 | 278 (308) | 90.26 | 213 (230) | 92.61 | 1853 (2160) | 85.79 |
| The worker(s) cared about the participants. | 1088 (1296) | 83.95 | 302 (329) | 91.79 | 276 (306) | 90.20 | 215 (232) | 92.67 | 1881 (2163) | 86.96 |
| The worker(s)' attitudes were very good. | 1076 (1295) | 83.09 | 307 (330) | 93.03 | 282 (307) | 91.86 | 213 (232) | 91.81 | 1878 (2164) | 86.78 |
| The worker(s) had much interaction with participants. | 1072 (1296) | 82.72 | 289 (327) | 88.38 | 268 (308) | 87.01 | 207 (233) | 88.84 | 1836 (2164) | 84.84 |
| On the whole, participants were satisfied with the worker(s). | 1122 (1298) | 86.44 | 312 (330) | 94.55 | 281 (308) | 91.23 | 215 (233) | 92.27 | 1930 (2169) | 88.98 |

The second observation is that only seven schools did not use the two dominant program elements in their Tier 2 Program, but most of the ratings were the highest among all different types of programs. It must be pointed out that unlike the Type A and Type B programs, Type D only involved students, and no teachers and parents were involved. Also, the average number of student participants in the Type D program was very low (37), while the number for Types A, B, and C were 65.12, 53, and 76.71, respectively (see Table 1). On the other hand, the high ratings may be due to the small sample size, i.e., a total of 259 students from seven schools. Also, because diverse activities were included in the Type D program, such as promoting discipline through military march training, social skills training, promoting learning skills and motivation, promoting self-understanding, and community-based oral history projects, further qualitative studies are necessary to illuminate the reasons for higher program effectiveness and to examine the correlation between program elements and program effectiveness.

Third, overall speaking, over 80% of participants were satisfied with the services received (see Table 2). Comparing the four types of programs, Type A received the lowest ratings in all the eight indicators for program satisfaction. However, only four indicators were rated positively below 80%. They are (1) "participants could get the service they wanted" (77.99%), (2) "the service provided could meet the participants' needs" (78.84%), (3) "the quality of the service was high" (79.41%), and (4) "participants would recommend others who have similar needs to participate in the program" (79.09%). Also, the last indicator "on the whole, participants were satisfied with the service" was rated positively by 84.37% of 1,292 participants. It revealed that Type A programs in different schools were well received.

**TABLE 4**
**Comparison of the Positive Views Towards Tier 2 Program Effectiveness Among Different Program Approaches**

| | Participants with Positive Responses in Different Program Approach | | | | | | | | | |
|---|---|---|---|---|---|---|---|---|---|---|
| | ABC Approach and Volunteer Training and Services | | ABC Approach | | Volunteer Training and Services | | Others | | Overall | |
| | N (Total Response) | % | N (Total Response) | % | N (Total Response) | % | N (Total Response) | % | N (Total Response) | % |
| The service has helped participants a lot. | 1016 (1280) | 79.38 | 291 (330) | 88.18 | 258 (300) | 86.00 | 208 (231) | 90.04 | 1773 (2141) | 82.81 |
| The service has enhanced participants' growth. | 1068 (1276) | 83.70 | 294 (330) | 89.09 | 265 (300) | 88.33 | 209 (229) | 91.27 | 1836 (2135) | 86.00 |
| In the future, participants would receive similar service(s) if needed. | 1012 (1277) | 79.25 | 284 (329) | 86.32 | 260 (300) | 86.67 | 210 (230) | 91.30 | 1766 (2136) | 82.68 |
| Participants have learned how to help themselves through participating in the program. | 1062 (1277) | 83.16 | 296 (327) | 90.52 | 266 (300) | 88.67 | 211 (227) | 92.95 | 1835 (2131) | 86.11 |
| Participants have had positive change(s) after joining the program. | 1046 (1274) | 82.10 | 290 (329) | 88.15 | 259 (299) | 86.62 | 215 (229) | 93.89 | 1810 (2131) | 84.94 |
| Participants have learned how to solve their problems through participating in the program. | 1053 (1276) | 82.52 | 299 (330) | 90.61 | 263 (296) | 88.85 | 208 (226) | 92.04 | 1823 (2128) | 85.67 |
| Participants' behavior has become better after joining this program. | 1015 (1275) | 79.61 | 275 (328) | 83.84 | 252 (300) | 84.00 | 210 (228) | 92.11 | 1752 (2131) | 82.21 |
| Those who knew the participants agree that this program has induced positive changes in them. | 996 (1274) | 78.18 | 271 (328) | 82.62 | 253 (299) | 84.62 | 201 (230) | 87.39 | 1721 (2131) | 80.76 |

Fourth, over 80% of participants rated positively on the eight indicators for the Tier 2 Program workers, ranging from 84.84% on "the worker(s) had much interaction with participants" to 88.98% on "on the whole, participants were satisfied with the worker(s)" (see Table 3). Comparing the four types of programs, Type A received the lowest ratings in all the eight indicators for program workers. However, all the ratings were positively over 80%, ranging from 82.24% on "the worker(s) understood the needs of the participants" to 86.44% on "on the whole, participants were satisfied with the worker(s)". It revealed that the social workers in different schools provided quality services. There are several possible reasons for the comparatively low ratings: (1) 60% of all participants joined the Type A program, covering a more diverse client population with different needs; (2) some adults were involved as participants — three schools with parents and two schools with both parents and teachers; (3) the average program attendance was among the highest, ranging from 83.34 to 86.63% (see Table 1).

Fifth, over 80% of participants rated positively on the eight indicators for program effectiveness, i.e., ranging from 80.76 to 86.11% (see Table 4), showing that the participants were generally satisfied with the results. Since the participants were generally satisfied with the program delivery and the workers, it is not surprising to see that the perceived program effectiveness was high. However, participants in the "others" category perceived the program to be more effective than the other three types of programs.

It is noteworthy that there are several limitations of the present study. First, as only Secondary 1 (i.e., Grade 7) students were involved, further studies are needed to examine each program type in order to illuminate issues of program effectiveness and whether or not adult participants rated differently in comparison with Secondary 1 participants. Second, only 70.74% of the program participants responded to Form C and some of the adult participants might not have completed the evaluation questionnaire. Obviously, effort should be made in order to understand the factors contributing to this observation. Third, as the present findings were "reconstructed" from the evaluation reports submitted by the agencies, the units of analyses were schools instead of individuals. As such, the power of the statistical analyses would become low and individual variations lost in the process. Finally, the limitations of subjective outcome evaluation utilizing quantitative findings should be realized[2,19] and addition of qualitative findings would be helpful[20,21]. Furthermore, if resources permit, studies attempting to examine the convergence of objective outcome evaluation findings and subjective outcome evaluation findings[22] should be carried out.

### ACKNOWLEDGMENTS

The preparation for this paper and the Project P.A.T.H.S. were financially supported by the Hong Kong Jockey Club Charities Trust.
